# Association between vertebral cross-sectional area and lumbar lordosis angle in adolescents

**DOI:** 10.1371/journal.pone.0172844

**Published:** 2017-02-28

**Authors:** Tishya A. L. Wren, Patricia C. Aggabao, Ervin Poorghasamians, Thomas A. Chavez, Skorn Ponrartana, Vicente Gilsanz

**Affiliations:** 1 Department of Orthopaedic Surgery, Children’s Hospital Los Angeles, Keck School of Medicine, University of Southern California, Los Angeles, California, United States of America; 2 Departments of Radiology, Children’s Hospital Los Angeles, Keck School of Medicine, University of Southern California, Los Angeles, California, United States of America; 3 Department of Pediatrics, Children’s Hospital Los Angeles, Keck School of Medicine, University of Southern California, Los Angeles, California, United States of America; Universidad de Zaragoza, SPAIN

## Abstract

Lumbar lordosis (LL) is more prominent in women than in men, but the mechanisms responsible for this discrepancy are poorly defined. A recent study indicates that newborn girls have smaller vertebral cross-sectional area (CSA) when compared to boys—a difference that persists throughout life and is independent of body size. We determined the relations between vertebral cross-sectional area (CSA) and LL angle and whether sex differences in lumbar lordosis are related to sex differences in vertebral CSA. Using multi-planar magnetic resonance imaging (MRI), we measured vertebral cross-sectional area (CSA) and vertebral height of the spine of 40 healthy boys and 40 girls, ages 9–13 years. Measures of the CSA of the lumbar vertebrae significantly differed between sexes (9.38 ± 1.46 vs. 7.93 ± 0.69 in boys and girls, respectively; P < 0.0001), while the degree of LL was significantly greater in girls than in boys (23.7 ± 6.1 vs. 27.6 ± 8.0 in boys and girls, respectively; P = 0.02). When all subjects were analyzed together, values for LL angle were negatively correlated to vertebral CSA (r = -0.47; P < 0.0001); this was also true when boys and girls were analyzed separately. Multivariate regression analysis indicated that vertebral CSA was independently associated with LL, even after accounting for sex, age, height or vertebral height, and weight. Similar negative relations were present when thoracic vertebrae were analyzed (Model P < 0.0001, R^2^ = 0.37, thoracic vertebral CSA slope P < 0.0001), suggesting that deficient vertebral cross-sectional dimensions are not merely the consequence of the anterior lumbar curvature. We conclude that vertebral CSA is negatively associated with LL, and that the greater degree of LL in females could, at least in part, be due to smaller vertebral cross-sectional dimensions. Studies are needed to examine the potential relations between vertebral CSA and spinal conditions known to be associated with increased LL, such as spondylolysis and spondylolisthesis.

## Introduction

Lumbar lordosis (LL) refers to the anterior curvature of the lumbar spine, expressed in humans as a response to upright posture and bipedalism. Accumulating evidence indicates that LL is present in early childhood, increases during adolescence [1], and is more prominent in women than in men [2,3,4,5,6], but neither the reasons nor the mechanisms responsible for this discrepancy are well-defined. A proposed hypothesis for sex differences in vertebral size is that it improves maternal performance in posture and locomotion. Pregnant women habitually extend their lumbar spine to counteract the shift in the center of mass associated with increases in abdominal size and weight by as much as 30% (~7 kg) [7,8,9,10]. Notably, the degree of lumbar lordosis in women is significantly associated with the number of pregnancies [8].

Although variations in lumbar vertebral morphogenesis, such as vertebral wedging and orientation of lumbar facets, have been suggested to facilitate lumbar lordosis [9,11,12], the structural basis for the greater degree of lumbar lordosis in females when compared to males are unclear. Recent data indicate that factors related to sex have a significant effect on intrauterine development of the axial skeleton [13]. Newborn girls have approximately 11% smaller vertebral cross-sectional area (CSA) when compared to boys [13]–a sexual dimorphism that increases further throughout childhood and is greatest at sexual and skeletal maturity [14,15,16]. Since the smaller female vertebra is associated with greater range of spinal motion [17], it could facilitate the lordosis needed to maintain upright posture during pregnancy [12].

Support for the notion that the sexual dimorphism in vertebral size in humans could represent an evolutionary adaptation to bipedal obstetric load would come from demonstrating an association between vertebral CSA and LL angle. In the current study, we examined the degree of LL and vertebral cross-sectional dimensions in 40 boys and 40 girls, ages 9–13 years, and hypothesized that 1) adolescent girls have a greater degree of lumbar lordosis than boys, 2) the degree of lumbar lordosis is predicted by vertebral CSA, and 3) differences in lumbar lordosis between boys and girls are related to sex differences in vertebral CSA.

## Materials and methods

### Study subjects

The Institutional Review Board for clinical investigations at Children’s Hospital Los Angeles (CHLA) approved this study, which was compliant with the Health Insurance Portability and Accountability Act. Written informed assent and consent were obtained from all participants and their parents.

For the purpose of this study, we chose boys and girls with ages corresponding to the adolescent growth spurt (9 to 13 years) and in early puberty when LL development markedly increases [4]. Participants were recruited from the AltaMed and Teenage and Young Adult Health Clinics in the Division of General Pediatrics at CHLA. We selected subjects who had a normal physical examination and no history of neuromuscular or chronic disease or use of medication. Subjects were also excluded if they had vertebral or spinal cord anomalies, scoliosis, or experienced recurrent episodes of low back pain. Height and weight measurements were obtained with participants dressed in examination gowns or lightweight clothing, without shoes. Body mass index (BMI) percentile was calculated with the Centers for Disease Control growth charts. For this study, values for testicular volume and degree of breast development were used to determine the Tanner stage of sexual development [18]. Only adolescents in early puberty (defined as Tanner stages 2 and 3) were included.

### MRI determinations

**Fig 1:**
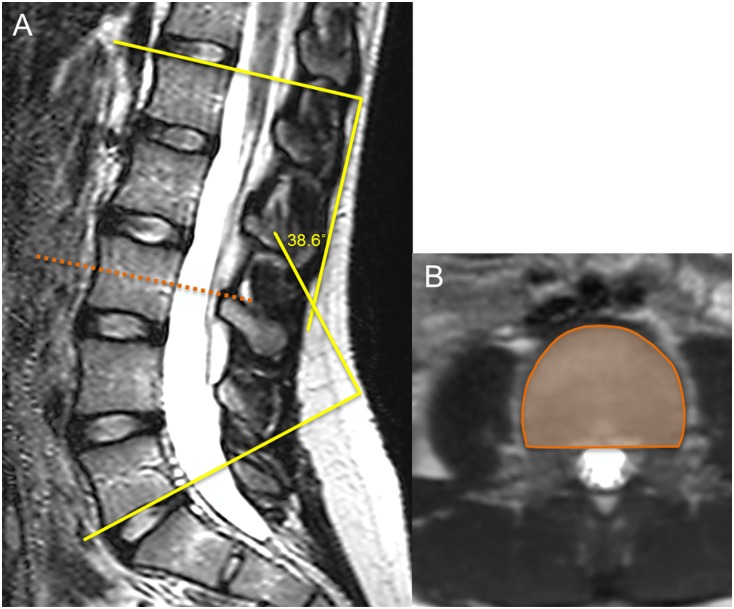
MRI images of the lumbar spine in a 13-year-old girl. (A) Coned-down sagittal image of the lumbar spine showing the degree of lumbar lordosis measured as the angle between the superior endplate of L1 and the inferior endplate of L5. (B) Axial image outlining the measurement of vertebral CSA at the third lumbar vertebra.

All MRI examinations were performed in the supine position with extended legs and without the use of general anesthesia or contrast enhancement. Subjects were examined using a research-dedicated 3.0 Tesla whole-body MRI scanner (Achieva R3.2, Philips Healthcare, Cleveland, Ohio) with a standard 15-channel spine coil. Three dimensional T2-weighted turbo spin echo scans were taken with TE of 120 ms, TR of 3000 ms, a flip angle of 90°, and with a voxel size of 1.0 x 1.0 x 1.0 mm. For the purpose of this study, LL was measured as the angle between the superior endplate of L1 and the inferior endplate of L5 (Fig 1A) [19], and vertebral CSA was measured in the axial plane at the midportion of the vertebral body based on the anterior and posterior heights from T10 to L5 (Fig 1B). Vertebral height was measured as the average of the anterior, posterior and midportion heights in the sagittal plane of the lumbar vertebrae. All measurements were analyzed offline with image processing software (Osirix; Pixmeo, Switzerland). The coefficients of variation for repeated MRI measurements of LL angle, vertebral CSA, and vertebral height are between 0.8–3.0% [20].

### Data analysis

The data were analyzed with the t-test for unpaired samples, and multivariate regression analysis was used to determine the association between vertebral CSA and LL. Collinearity among predictor variables was considered a tolerance level < 0.01 or variance inflation factor > 10. Confounding variables were considered a > 10% change in the CSA slope. Multivariate regression assumptions (linearity, independence, normality and homoscedasticity) were ascertained. Continuous variables are presented as mean ± SD unless otherwise indicated. Statistical significance was considered p < 0.05. Data was analyzed using SAS (Statistical Software Package v 9.4, Cary, NC).

## Results

The characteristics of study subjects are shown in Table 1. Boys were significantly older and heavier, and tended to be taller when compared to girls (Table 1). There were, however, no sex differences in height or weight percentiles between sexes.

**Table 1:** Ages, anthropometric characteristics, and MRI measures of vertebral morphology and degree of lumbar lordosis in 80 healthy adolescent boys and girls.

	Males (*n = 40*)	Females (*n = 40*)	P value
Age (yr)	11.5 ± 1.35	10.8 ± 1.21	0.015
Height (cm)	149.2 ± 11.2	145.3 ± 8.34	0.083
Height (%)	59.4 ± 28.7	58.9 ± 26.3	0.939
Weight (kg)	43.8 ± 12.6	38.3 ± 8.5	0.025
Weight (%)	58.6 ± 32.0	54.6 ± 32.0	0.577
BMI (kg/m^2^)	19.5 ± 4.59	18.0 ± 2.98	0.081
Vert. height (cm)	1.94 ± 0.21	1.95 ± 0.18	0.734
Vert. CSA (cm^2^)	9.38 ± 1.46	7.93 ± 0.73	<0.0001
Lumbar lordosis (°)	23.7 ± 6.05	27.6 ± 8.04	0.018

As expected, measures of the CSA of the lumbar vertebrae significantly differed between sexes (Table 1); on average, vertebral CSA was 15.5% smaller in girls. Measures of vertebral height were comparable between sexes, suggesting that the mild sex differences in standing heights were due to leg lengths. In contrast, the degree of LL was significantly greater in girls than in boys; on average, the angle of lordosis was approximately 16.5% greater in girls (Table 1).

When all subjects were analyzed together, there were no associations between lordosis and age, weight, or standing or vertebral height (all r’s ≤ 0.11; all P’s ≥ 0.32). In contrast, values for LL angle were negatively correlated to vertebral CSA (r = -0.47; P < 0.0001); this was also true when boys and girls were analyzed separately (r’s = -0.53 and -0.33 in boys and girls, respectively; both P’s ≤ 0.04) (Table 2).

**Table 2:** Correlation matrix of age, anthropometric characteristics, and MRI measures of vertebral morphology and degree of lumbar lordosis in 40 healthy adolescent boys and 40 adolescent girls.

	Age (yr)	Height (cm)	Weight (kg)	BMI (kg/m^2^)	Vert. height (cm)	Vert. CSA (cm^2^)	Lumbar lordosis (°)	
Age (yr)		0.72^c^	0.47^b^	0.14	0.68^c^	0.43^a^	-0.16	Boys
Height (cm)	**0.54**^c^		0.61^c^	0.11	0.84^c^	0.67^c^	-0.29	
Weight (kg)	**0.25**	**0.72**^c^		0.85^c^	0.65^c^	0.64^c^	-0.21	
BMI (kg/m^2^)	**-0.03**	**0.30**	**0.88**^c^		0.27	0.34^a^	-0.06	
Vert. height (cm)	**0.67**^c^	**0.86**^c^	**0.70**^c^	**0.37**^a^		0.62^c^	-0.27	
Vert. CSA (cm^2^)	**0.07**	**0.31**^a^	**0.40**^a^	**0.35**^a^	**0.28**		-0.53^c^	
Lumbar lordosis (°)	**0.49**^b^	**0.26**	**0.27**	**0.18**	**0.43**^b^	**-0.33**^a^		
	**Girls**							

^a^P ≤ 0.05

^b^P ≤ 0.01

^c^P ≤ 0.001

Multivariate regression analysis indicated that sex was independently associated with LL, even after accounting for age, height or vertebral height, and weight (Table 3a). The addition of vertebral CSA as an independent variable increased the predictive power of the model (R^2^ increased from 0.14 to 0.37), but eliminated the contribution of sex (Table 3b).

**Table 3:** Multivariate regression models for the prediction of lumbar lordosis without (A) and with (B) the inclusion of vertebral CSA.

	Variable	Parameter Estimate ± SE	P value
**A.**	Sex	0.321 ± 1.646	0.006
	Age (yr)	0.353 ± 0.810	0.018
	Height (cm)	-0.283 ± 0.127	0.108
	Weight (kg)	0.066 ± 0.096	0.653
	*Model P = 0*.*02*, *F-Value*: *3*.*01*, *R*^*2*^: *0*.*138*		
**B.**	Sex	0.191 ± 1.654	0.849
	Age (yr)	2.388 ± 0.701	0.020
	Height (cm)	-0.262 ± 0.115	0.794
	Weight (kg)	2.191 ± 0.088	0.032
	Vertebral CSA (cm^2^)	-0.728 ± 0.761	<0.0001
	*Model P <0*.*0001*, *F-Value*: *8*.*56*, *R*^*2*^: *0*.*366*		

When lumbar vertebral CSA was replaced with the mean vertebral CSA of the low thoracic spine (T10–T12), the negative relationship persisted (Model P < 0.0001, R^2^ = 0.37, T10–T12 vertebral CSA slope P < 0.0001).

## Discussion

Lumbar lordosis is a likely adaptation to ensure stability when standing and walking, yet the structural basis for anterior lumbar curves and the reasons for their greater prominence in females are unclear. In this study, we examined the potential relations between vertebral cross-sectional dimensions and LL, and found that young females have significantly smaller vertebral CSA and a greater degree of lumbar curvature than young males. We also found a significant negative correlation between vertebral CSA and the degree of LL, which was present in both sexes and independent of age, weight, and standing or vertebral height. Moreover, regression analysis indicated that differences in LL between boys and girls were closely related to sex differences in vertebral CSA. Notably, the association between vertebral CSA and LL angle was also present when the cross-sectional dimensions of the thoracic rather than lumbar vertebrae were analyzed. These findings suggest that deficient vertebral growth is not merely the consequence of exaggerated anterior spinal curve, but likely a structural determinant of LL in adolescents.

These findings have particular relevance to women during pregnancy, since smaller vertebrae would facilitate the anterior lumbar curvature needed to compensate for the obstetric load and maintain bipedal posture. They may also offer a new potential predictor of general spinal health. Lumbar spondylolysis is a condition specific to the unique human anatomical characteristic of LL [21] and a common cause of back pain in adolescents [22,23]. Accumulating evidence indicates that greater lordosis angle is a risk factor for developing spondylolysis and ventral slippage of the affected vertebra [24,25,26,27,28,29,30,31,32,33,34]. However, the degree to which LL is associated with low back pain has been a matter of considerable debate [35]. While some studies suggest that patients with chronic low back pain have increased LL compared to controls [36,37], others found no such relation [38,39,40,41]. Studies are needed to establish whether variations in vertebral cross-sectional growth influence the susceptibility for low back pain, spondylolysis, and spondylolisthesis.

This study has other notable limitations, including LL measurement method and the population examined. All subjects had to be analyzed in the supine position using a uniform method of evaluation for LL; prior data has shown that LL in the upright position can be reproduced by positioning the patient supine with straightened lower extremities [42]. Additionally, our analysis was confined to healthy adolescent boys and girls, but whether our results are translatable to adult populations is unknown. Since the sexual dimorphism in vertebral size increases during puberty and is greatest at sexual and skeletal maturity when young women have ~25% smaller vertebral CSA, even after considering differences in body size [14,15,16], the strong relations between vertebral CSA and LL angle are likely translatable to adulthood.

Although we did not have direct measurements of spine flexibility for the children, studies suggest a positive correlation between the degree of LL and lumbar spinal motion [43,44]. We propose that the relationship between smaller vertebral CSA and increased LL is mediated by increased spinal flexibility [17], though the relationship may also be affected by other factors such as loading in the lumbar region. We also acknowledge that we did not control for other potential determinants of lumbar lordosis and flexibility, such as intervertebral disc and apophyseal morphology, paraspinous musculature, and ligamentous support. These anatomical traits are difficult to assess and/or have a great range in variability, even within the same individual [45,46].

In conclusion, understanding sexual dimorphisms is relevant to many fields of medicine and must also be considered in this discussion of lumbar lordosis. Upright posture presents a unique set of challenges to the pregnant female spine. The current study provides new evidence that vertebral CSA is a structural determinant of LL, and that the greater degree of LL in females could, at least in part, be due to smaller vertebral cross-sectional dimensions. Studies are needed to corroborate and establish the generalizability of our results in other populations, and to explore the potential relations between vertebral CSA and low back pain and spinal conditions known to be associated with increased LL, such as spondylolysis.

## Supporting information

S1 Table Ages, anthropometric characteristics, and MRI measures of vertebral morphology and degree of lumbar lordosis included in the analyses. (XLSX)
